# Precision Medicine: The Role of the MSIDS Model in Defining, Diagnosing, and Treating Chronic Lyme Disease/Post Treatment Lyme Disease Syndrome and Other Chronic Illness: Part 2

**DOI:** 10.3390/healthcare6040129

**Published:** 2018-11-05

**Authors:** Richard I. Horowitz, Phyllis R. Freeman

**Affiliations:** 1 HHS Tickborne Disease Working Group, Washington, DC 20201, USA; 2 Hudson Valley Healing Arts Center, New York, NY 12538, USA; freemanp63@gmail.com

**Keywords:** Chronic Lyme disease, Post Treatment Lyme Disease Syndrome (PTLDS), dapsone, Multiple Systemic Infectious Disease Syndrome (MSIDS), persister bacteria, precision medicine, Chronic Variable Immune Deficiency (CVID), Postural Orthostatic Tachycardia Syndrome (POTS)

## Abstract

We present a precision medical perspective to assist in the definition, diagnosis, and management of Post Treatment Lyme Disease Syndrome (PTLDS)/chronic Lyme disease. PTLDS represents a small subset of patients treated for an erythema migrans (EM) rash with persistent or recurrent symptoms and functional decline. The larger population with chronic Lyme disease is less understood and well defined. Multiple Systemic Infectious Disease Syndrome (MSIDS) is a multifactorial model for treating chronic disease(s), which identifies up to 16 overlapping sources of inflammation and their downstream effects. A patient symptom survey and a retrospective chart review of 200 patients was therefore performed on those patients with chronic Lyme disease/PTLDS to identify those variables on the MSIDS model with the greatest potential effect on regaining health. Results indicate that dapsone combination therapy decreased the severity of eight major Lyme symptoms, and multiple sources of inflammation (other infections, immune dysfunction, autoimmunity, food allergies/sensitivities, leaky gut, mineral deficiencies, environmental toxins with detoxification problems, and sleep disorders) along with downstream effects of inflammation may all affect chronic symptomatology. In part two of our observational study and review paper, we postulate that the use of this model can represent an important and needed paradigm shift in the diagnosis and treatment of chronic disease.

## 1. Introduction

Lyme disease is rapidly spreading and there has been a 320% increase in the number of US counties affected with Lyme disease within the past 20 years [1], with another recent threefold increase in the number of vector-borne disease cases [2]. Among the almost 650,000 vector-borne reported cases during a 12-year period, over 491,000 were tick-borne and over 150,000 were mosquito-borne [3]. According to prior National Institutes of Health (NIH) studies, those patients with symptoms of chronic Lyme disease, both diagnosed and undiagnosed, are extremely ill [4] and many are disabled and unable to work [5].

Nearly one out of two adults in the United States during the past decade have been found to suffer from at least one chronic condition [6], with estimates of US disability rates ranging from 13–19% [7]. Some of these disabling symptoms include arthritis, pain, fatigue, and cognitive difficulties. These are hallmark symptoms of Lyme disease and associated co-infections. Since the present Lyme disease diagnostic two-tiered testing strategy used for surveillance purposes [8] is known to have a sensitivity/specificity averaging around 56% [9], and as per the Centers for Disease Control and Prevention (CDC), the surveillance case definitions “are not intended to be used by health care providers for making a clinical diagnosis …” [10], approximately half of the individuals with Lyme disease may go undiagnosed. Some individuals suffering from “medically unexplained symptoms” (MUS) may therefore have contracted a tick-borne illness. Inadequate diagnostic testing for Lyme disease and associated *Borrelia* species may also be contributing to this diagnostic dilemma, as discussed in detail in Precision Medicine: Retrospective Chart Review and Data Analysis of 200 Patients on Dapsone Combination Therapy for Chronic Lyme Disease/PTLDS: Part 1. There has been an expansion of other *Borrelia sensu lato* species across the United States in the past decade [11], and many of these borrelia species, including Relapsing Fever *Borrelia* spp. and *Borrelia miyamotoi* as well as *Borrelia bissetti*, will not be found on standard two-tiered testing strategies for Lyme disease, yet they can lead to unexplained chronic illness [12,13,14,15]. 

The true healthcare burden for tick-borne illness may also be unappreciated due to Lyme disease and coinfections mimicking other chronic illnesses. Five percent of the US population suffers from Chronic Fatigue Syndrome (CFS)/Myalgic Encephalomyelitis and Fibromyalgia [16], which share the same symptoms as Lyme disease. The diagnostic criteria for these two diseases are based on symptomatology and establishing a differential diagnosis, not on specific laboratory testing. The true number of individuals with borreliosis and co-infections resulting in these chronic fatiguing, musculoskeletal illnesses with cognitive difficulties is therefore unknown. Prior reports on the role of *Bartonella* species indicate, for example, that this class of bacteria can cause a broad range of rheumatologic and neurologic symptoms resembling CFS/fibromyalgia [17,18,19].

Spirochetes have also been reported to be found in the brains of individuals suffering from dementia, and in the biofilms of patients suffering from Alzheimer’s disease [20,21]. Multiple scientific peer-reviewed journal articles in the past two decades have implicated a possible association between bacterial and viral infections [22] along with environmental toxins in neurodegenerative conditions, with recent healthcare estimates approximating that 46 million Americans presently suffer from pre-clinical dementia [23]. Environmental toxins and spirochetes have both been associated with cognitive difficulties, as well as autoimmune illness, which presently affects tens of millions of Americans [24]. The necessity of effective prevention, diagnostic, and treatment strategies for Lyme disease and associated co-infections, and the need to evaluate their role in these disorders is urgently needed based on the above statistics. Just as important, however, is the need to determine the role of overlapping infections, environmental toxins, and other etiologies increasing inflammation, resulting in diverse chronic disease manifestations. If we are to improve public health and control rising health care costs, a new paradigm to account for the rising burden of chronic illness is needed.

The etiology and treatment of chronic Lyme disease/Post Treatment Lyme Disease Syndrome (PTLDS) has been a hotly debated topic in the medical literature for the past three decades. This problem exists in part because of a lack of clear definitions. PTLDS is defined as a syndrome in patients who have been treated for an erythema migrans rash (EM) with appropriate antibiotic treatment who have “persistent or recurrent patient-reported symptoms of fatigue, musculoskeletal pain, and/or cognitive complaints with associated functional decline, and this syndrome represents a defined subset of the larger patient population with the diagnosis of chronic Lyme disease, which is less understood and well defined” [25,26]. Theories of why patients remain ill generally range from autoimmune reactions post infection to tissue damage and/or persistent infection of the spirochete and/or its parts. No one model, however, has been sufficient to explain ongoing symptomatology after standard courses of antibiotics. The prevailing medical model used to describe and explain most chronic infectious disease is the “one microorganism/one disease” model based on Koch’s postulates taught in medical school. This theory was established in the late 1800s. Scientific advances since that time include significant improvements in diagnostics as well as identifying expanding tick populations with a better understanding of the tick microbiome and associated coinfections, along with identifying the role of borrelia, other intracellular bacteria (i.e., *Bartonella* spp. and *Mycoplasma* spp.), the gastrointestinal (G.I). microbiome, and environmental toxin exposures in autoimmune illness. The role of nutritional deficiencies, food allergies/sensitivities, leaky gut [27], and/or sleep disorders, which can contribute to free radical/oxidative stress and further increase inflammation and symptomatology [28,29,30,31,32,33,34,35,36,37,38,39], have also been identified in the recent medical literature as potential etiological causes of chronic symptoms. 

All these factors can have deleterious downstream effects on the body, including, but not limited to, mitochondrial and liver dysfunction; Hypothalamic-Pituitary-Adrenal (HPA) axis and autonomic nervous system dysfunction; as well as the ability to increase neuropsychiatric symptoms and pain syndromes [40,41]. The establishment of a new paradigm to account for all these factors and their roles in causing disabling symptoms after standard treatment for chronic Lyme disease/PTLDS is of vital importance based on the significant numbers of individuals contracting vector borne diseases. A data mining approach in a large cohort of symptomatic Lyme disease patients was undertaken to be able to better define the role of these multiple variables in those suffering from resistant symptoms of chronic Lyme disease/PTLDS. 

In 2012, Horowitz described a multifactorial model for chronic disease known as MSIDS, or Multiple Systemic Infectious Disease Syndrome [39]. The individual patient’s risks are evaluated during the initial evaluation as the model recognizes that a “one size fits all” approach using general medical guidelines may not account for individual differences and risk factors. The 16-point MSIDS model can efficiently screen through multifactorial etiologies contributing to chronic illness and focus on prevention (epigenetics), thus personalizing treatment. It represents a potential paradigm shift in the diagnostic and treatment approaches for chronic disease, as no one factor is assumed in advance to play a predominant role in the patient’s symptomatology. It is only after taking a detailed history, evaluating chief complaints, reviewing family, social, and environmental histories, checking a review of systems, and performing a physical examination that medical hypotheses are formed, leading to focused laboratory testing.

Factors on the 16-point MSIDS model [42], which are then evaluated based on the history and physical examination, and can contribute to chronic disease include: 

(1) **Infections**: Four types of infections are assessed. Some are tickborne and others may be mosquito borne, and/or transmitted by other vectors (including fleas, lice, mites, biting flies, and spider bites) or due to human to human transmission: 

(a) Bacteria: i.e., *Borrelia burgdorferi* [Lyme disease]; other *Borrelia* species, such as *Borrelia sensu lato* species [43] and Relapsing Fever; *Ehrlichia*, *Anaplasma*, *Bartonella* species; *Mycoplasma* and *Chlamydia* species; *Rickettsia* species [*Rickettsia rickettsia* (Rocky Mountain spotted fever), *Coxiella burnetii* (Q fever), *Rickettsia typhi* (typhus)]; *Francisella tularensis* [tularemia]; and *Brucella* spp. [Brucellosis].

(b) Parasites: *B. microti* and *B. duncani* [Babesiosis], other piroplasms, *Toxoplasma gondii (*toxoplasmosis*)*, intestinal parasites.

(c) Viruses: Herpes simplex virus 1 (HSV1), Herpes simplex virus 2 (HSV2), Human Herpes Virus 6 (HHV-6), Epstein Barr Virus [EBV], Cytomegalovirus [CMV], Coxsackie virus, Parvovirus, West Nile virus (WNV).

(d) Candida and other fungi.

(2) **Immune dysfunction**: *Borrelia burgdorferi* as well as European strains, including *Borrelia garinii*, have been associated with autoimmune phenomena [44,45]. Autoimmune markers, including antinuclear antibodies (ANA) and rheumatoid factors (RF), were assessed, as well as Human Leukocyte Antigen (HLA) markers (DR2, DR4) and immunoglobulin deficiencies and/or subclass deficiencies [46].

(3) **Inflammation**: Inflammatory chemokines and cytokines are produced during infection [47,48,49]. Erythrocyte Sedimentation Rate (ESR), C-Reactive Protein (CRP, an indirect marker of IL-6), Human Transforming Growth Factor beta 1 (TGFB1), Complement component 3a (C3a), Complement component 4a (C4a), and Vascular Endothelial Growth Factor (VEGF), an indirect marker of *Bartonella* infection [30]) were gauged as markers of inflammation.

(4) **Toxicity**: The burden of heavy metals, including mercury, lead, arsenic, cadmium, and aluminum, were recorded [50,51,52] as well as levels of mold toxins, including aflatoxins, trichothecenes, ochratoxins, and gliotoxins. Neurotoxins, such as quinolinic acid, may also be produced during infection [53,54]. Patients with a history of multiple chemical sensitivity (MCS) and/or Parkinson’s disease were evaluated for the presence of pesticides, as well as a clinical response to intravenous and oral glutathione, which is known to play a role in chemical detoxification [55].

(5) **Allergies/Sensitivities**: Foods [56,57], medications, and environmental allergies were recorded. Inflammatory cytokine production, similar to those produced during a Lyme infection, may be found in those with allergic reactions. Markers, including total IgE antibody levels, IgE food allergies, evidence of gluten sensitivity or celiac disease (antigliadin antibodies, tissue transglutaminase (TTG)), and histamine levels were recorded if pruritis and/or symptoms of Mast Cell Activation Disorder were present [58]. 

(6) **Nutritional and enzyme deficiencies/functional medicine abnormalities in biochemical pathways** [59,60]: Patients with poor nutritional intake were tested for amino acid and/or fatty acid deficiencies. All patients were checked for methylenetetrahydrofolate reductase (MTHFR) gene mutations as well as mineral deficiencies, including iodine, copper, zinc, and magnesium. These minerals are essential cofactors in the biochemical pathways responsible for controlling free radical/oxidative stress, inflammation, hormone production, and detoxification.

(7) **Mitochondrial dysfunction [61,62,63]**: The mitochondria are essential for energy production in the muscles, nerves, brain, liver, kidney, and heart. Mitochondrial dysfunction was defined as those who had positive responses to the following mitochondrial support supplements: NT Factors, i.e., glycosylated phospholipids, CoQ10, acetyl-l-carnitine, and d-ribose.

(8) **Psychological disorders**: Neuropsychiatric symptoms may result from and/or worsen when Lyme disease and associated coinfections, such as *Bartonella* spp. and *Babesia* spp., are present [64,65]. Common manifestations, including depression, anxiety, Obsessive Compulsive Disorder (OCD), and Post Traumatic Stress Disorder (PTSD), were recorded [66]. 

(9) **Neurological dysfunction**: An infection with *Borrelia burgdorferi* and associated coinfections, including, but not limited to, other *Borrelia* species, *Babesia* spp., *Bartonella* spp., *Rickettsia* spp., and *Mycoplasma* spp., may increase neurological dysfunction [20,47,67]. We evaluated patients using our online symptom survey for evidence of neurological symptoms (headaches, cognitive dysfunction, as well as neuropathy). Proof of neuropathy with or without Chronic Inflammatory Demyelinating Polyneuropathy (CIDP) were also determined by direct chart review of physical examination/electromyogram (EMG)/small fiber biopsies.

(10) **Endocrine disorders [68,69,70]**: Hypothalamic-Pituitary-Axis (HPA) axis dysfunction may result from an infectious process. Evidence of thyroid, adrenal, and sex hormone dysfunction were recorded. Adrenal function was measured by blood, urine, and saliva testing [71]. Vitamin D levels (whose ratios can be an indirect marker for the presence of intracellular infections), as well as precursors of hormones, including Dehydroepiandrosterone sulfate (DHEA-S) and pregnenolone, were also noted.

(11) **Sleep disorders** [72,73]: Lyme disease is known to result in circadian rhythm disorders [74], including delayed sleep phase syndrome (DSPS) [75], where patients have a challenging time falling asleep and/or staying asleep. Hypersomnolence due to inflammatory cytokine production is also a known clinical manifestation [76,77,78]. Individuals were evaluated for evidence of any sleep related disorders, including obstructive sleep apnea (OSA), restless leg syndrome (RLS), hormone imbalance (menopause, elevated adrenal function at night), benign prostatic hypertrophy (BPH), and/or medication induced sleep problems.

(12) **Autonomic nervous system (ANS) dysfunction and Postural Orthostatic Tachycardia Syndrome (POTS)** [79,80,81,82,83,84]: Postural orthostatic tachycardia syndrome following Lyme disease has been reported [85], and four principal types of neuropathy can affect the nervous system in the patient infected with *Borrelia burgdorferi*. Autonomic neuropathy is a form of polyneuropathy that affects elements of the central nervous system (brain/hypothalamus and spinal cord), peripheral nervous system with its sensory motor branches, and the enteric nervous system made up of nerve fibers that go to the bladder and gastrointestinal tract (including the pancreas and gallbladder). Problems with the autonomic nervous system can result in symptoms of resistant fatigue, dizziness, low blood pressure and fainting, anxiety, palpitations, cognitive difficulties, absent or excessive sweating, problems with temperature dysregulation [86], and problems with gastroparesis (nausea, vomiting) with or without constipation and/or bladder dysfunction. POTS is often diagnosed by a Head-up Tilt Table Test (HUT), but if such testing is not available, POTS can be diagnosed with bedside measurements of heart rate and blood pressure taken in the supine (laying down) and standing up position [87]. In our study, we performed sitting and standing blood pressures with corresponding pulse rates at time 0 (sitting for several minutes), 3, 6, and 9 min were recorded. Mild POTS was defined as a 1–10 mm Hg drop in blood pressure (BP), and/or 1–10-point increase in heart rate after standing for 9 min; moderate POTS was defined as a 11–29 mm drop in BP, and/or 11–29-point increase in heart rate after standing for 9 min; severe POTS was defined as a 30+ increase in heart rate standing and/or drop in systolic or diastolic blood pressure by 30 mm Hg or greater, standing for 9 min. More detailed tests to evaluate the autonomic nervous system [86], such as the Quantitative Sudomotor Axon Reflex Test (QSART), Heart Rate Response to deep breathing (HRDB), Valsalva maneuver (VM), Thermoregulatory Sweat Test (TST), Quantitative sensory testing (QST), skin biopsies evaluating the small fiber nerves, and gastric motility studies were performed in a small subset of patients with symptoms of severe autonomic neuropathy.

(13) **Gastrointestinal (G.I.) disorders** [27,88]: Certain G.I. disorders may result in increased inflammation, so patients were evaluated for one or more of the following gastrointestinal complaints: Gluten sensitivity, celiac disease, colitis, Candidiasis, leaky gut, parasites, *Helicobacter pylori* exposure, gastroesophageal reflux disease (GERD), and/or a history of *Clostridium difficile* while on dapsone combination therapy (DDS CT). Microbiome analysis and evaluation of beneficial short chain fatty acids (SCFA), inflammation, pancreatic enzymes, and fat malabsorption was done through Genova/Metametrix when clinically indicated.

(14) **Elevated liver function testing (LFT’s)**: Elevated liver functions have been associated with inflammatory cytokine production [89], and may result from Lyme disease and associated tickborne infections, including, but not limited to, anaplasmosis, ehrlichiosis, Rocky Mountain spotted fever, babesiosis, and relapsing fever (*Borrelia miyamotoi*). Other causes of elevated liver functions include alpha-1 antitrypsin deficiency, Wilson’s disease, hemochromatosis, viral and autoimmune hepatitis, gallstones, inflammatory bowel disease, connective tissue disease, congestive heart failure (right-sided), hepatic steatosis (Non Alcoholic Steatohepatitis, i.e., NASH), acute and chronic pulmonary disease, endocrine disorders, chemical and drug exposure, as well as cancer [90,91,92]. Evidence of elevated Aspartate Aminotransferase (AST), Alanine Aminotransferase (ALT), alkaline phosphatase, and total bilirubin were recorded. Patients were tested for the above liver pathologies if liver function abnormalities persisted.

(15) **Pain syndromes**: Muscular, arthritic, and neuropathic pain syndromes [93,94,95,96] (often migratory nature, which is one of the hallmarks of Lyme disease [96]) along with headaches/migraines can be seen with tick-borne disorders. Evidence of these syndromes were noted. Some patients were on compounded medications for pain and inflammation, including low dose naltrexone (LDN) and glutathione.

(16) **Physical Therapy** (**PT)/deconditioning** [97,98,99]: Many patients are deconditioned due to long-standing chronic illness. The need for physical therapy and reconditioning programs was evaluated along with their efficacy with improving fatigue, muscle strength, and coordination.

These numerous factors on the 16-point MSIDS model can be arranged into two categories (multiple causes of inflammation and the downstream effects of inflammation), which can account for persistent symptoms in tickborne and other chronic disease. The causes of inflammation include multiple infections, immune dysfunction, genetic causes of autoimmunity, imbalances of the microbiome of the gut, food allergies/sensitivities, leaky gut, mineral deficiencies and sleep disorders. There are also factors which have adverse downstream effects at both the cellular and organ systems levels, leading to resistant fatigue, pain, and neurocognitive symptoms. These would include potential downstream effects of inflammation leading to endocrine disorders (low testosterone, estradiol, and progesterone with low libido; low adrenal function; hypothyroidism), neurological and psychological dysfunction, POTS/dysautonomia, mitochondrial dysfunction, pain syndromes, liver dysfunction, and autoimmune phenomenon. Any of these chronic disease manifestations may be worsened and/or due to one or multiple factors. This is the case with autoimmune reactions, which the scientific literature has shown can be affected by exposure to borrelia and other infections [100] (including, but not limited to, *Bartonella* and *Mycoplasma* species) [30,32,33], environmental toxin exposure (i.e., mercury, bisphenol A, asbestos, and/or small particle pollution) [50,51,101,102], imbalances in the microbiome of the gut, and/or from a genetic predisposition [103]. We therefore collected data from an online survey, which evaluated the efficacy of dapsone combined with other antibiotics and agents that disrupt biofilm for the treatment of chronic Lyme disease/PTLDS, along with information data mined directly from 200 patient records detailing abnormalities on the MSIDS model.

In part one, we evaluated the efficacy of newer “persister” drug regimens, like dapsone combination therapy, and found this protocol decreased the severity of eight major Lyme symptoms and improved treatment outcome. We also found multiple species of intracellular bacteria, including *Rickettsia*, *Bartonella*, *Mycoplasma*, *Chlamydia*, *F. tularensis*, and *Brucella*, contributing to the burden of illness, as well as a high prevalence of *Babesia* complicating management, with probable geographic spread of *Babesia WA1/duncani* to the Northeast. Occasional reactivation of viral infections, including HHV-6, in immunocompromised individuals was also seen in a small percentage of patients. 

In part two, we seek to understand how Lyme disease can affect different body systems, how abnormalities on the MSIDS model can affect chronic symptoms in those with Lyme disease and associated coinfections, as well as which combination of factors might contribute to the burden of chronic illness leading to resistant symptomatology.

## 2. Materials and Methods

### 2.1. Participants

Participants included in this project were 200 adults recruited from a specialized Lyme disease medical practice using email and telephone contacts. Although situated in the Northeastern United States, the medical practice attracts patients from all over the world. Of 200 participants, 67 (33.5) were male, and 133 (66.5%) were female. Age ranged from 18–84 (M = 52.04, SD = 16.66). Out of 200 participants, 4 (2%) were Asian (Non-Hispanic), while the rest were all White (Non-Hispanic). Participants were mostly from the United States (N = 193), which was divided into demographic regions: West Coast (N = 1), Midwest (N = 16), East Coast (North) (N = 155), East Coast (South) (N = 20), and Other (Hawaii) (N = 1). Potential participants were sent an email invitation containing a link to the online symptom survey reported in Precision Medicine: Part 1.

### 2.2. Methodology

We conducted a retrospective chart review of a convenience sample of patients who agreed to have their medical charts reviewed. These charts (N = 657) were from the 200 patients who served as participants in our dapsone trial (reported in Part 1) and who had given informed consent to examine their medical records for MSIDS variables. Most of these patients had multiple charts documenting their treatment over the course of many years. Patients had enrolled in the preliminary dapsone trial based on the drug’s action on “persister” bacteria [104,105,106,107]. Each participant received detailed instructions that outlined the need for blood testing every three weeks, dietary guidelines, and the name and phone number of the medical center’s head nurse if anyone had questions or medical issues. Surveys were given in office, online, and via telephone to gather patient information.

**Inclusion criteria:** All 200 patients in our study met the criteria for a clinical diagnosis of Lyme disease supported by a physician documented erythema migrans (EM) rash and/or positive laboratory testing, including a positive ELISA/Enzyme Immunoassay (EIA), and/or C6 ELISA, Immunofluorescent Antibody (IFA), CDC positive IgM and/or IgG Western Blot, Polymerase Chain Reaction (PCR), *Borrelia* specific bands (23, 31, 34, 39, 83/93) on a Western blot [108] and/or positive ELISPOT (Lymphocyte Transformation Test, i.e., LTT). These patients had either failed or had an inadequate response to prior antibiotic therapy, and/or relapsed with persistent symptoms after stopping anti-infective therapy. 

**Exclusion criteria:** Patients under the age of 18, patients having a known allergy to dapsone or any medication used in the trial, and/or having significant laboratory abnormalities, including a pre-trial anemia, were excluded from our study. 

### 2.3. Data Mining Procedure

After we identified and operationally defined the MSIDS study variables we wished to explore, we provided those conducting the chart review with a list of these variables and their definitions, and provided them with a set of procedures to follow, including inclusion and exclusion criteria for each variable. Following the training of our data “miners” and a pilot test of the procedures, we provided them with oversight and closely monitored the procedure for consistency. All data mining was conducted via laptop computers using a standardized set of operationally defined variables recorded on duplicate Excel data sheets. Each research assistant on our team used his or her own Excel sheet and worked in pairs (one member entering data while the one member identified the variables in the chart). Excel sheet data were then combined/merged for data analysis. All data entry was from de-identified participants, and their PHI (personal health information) was entered via a research code assigned at random. Participant names and other identifiers were only known to the first author of this paper and the head nurse, who, with the first author, provided oversight to the procedures. All of those involved in data mining the charts and data analysis were HIPPA trained, had backgrounds and education in scientific data collection, and signed additional confidentiality agreements with our medical center. The first author was directly involved in data mining and data entry, and the second author provided oversight and team coordination. Data analysis of the Excel data was via SPSS software (version 25.0 from IBM SPSS Statistics, Armonk, NY, USA).

### 2.4. Laboratory Testing

Analysis of MSIDS variables were conducted using blood testing from several national reference laboratories (Quest Diagnostics, Secaucus, NJ, USA; LabCorp, Burlington, NC, USA; BioReference, Elmwood Park, NJ, USA; Pacific Toxicology Laboratories, Chatsworth, CA, USA), local state laboratories (i.e., Sunrise Medical Laboratories, Hicksville, NY, USA; NorDx, Scarborough, ME, USA; Affiliated Laboratory Inc., Rutland, VT, USA, AccuReference Medical Lab, Wappingers Falls, NY, USA), specialty laboratories for tick-borne diseases (Imugen, Norwood, MA, IgeneX, Palo Alto, CA, USA; Medical Diagnostic Laboratory, Hamilton Township, NJ, USA; Stony Brook Lyme Disease Laboratory, Stony Brook, NY, USA; Milford Molecular Diagnostics, Milford, CT, USA; Galaxy diagnostics, Morrisville, NC, USA; Immunosciences Lab, Inc, Los Angeles, CA, USA), and functional medicine laboratories (Aeron Lifecycles Clinical Laboratory, San Leandro, CA, USA; Labrix Clinical Laboratory, Clackamas, OR, USA; Genova Diagnostics, Asheville, NC, USA; Great Plains, Lenexa, KS, USA; Diagnos-Tech, Kent, WA, USA; Doctor’s Data, St Charles, IL, RealTime Laboratory, Carrollton, TX, USA). More than one laboratory was used for each patient depending on laboratory capability, patient insurance, and availability in their home state (Galaxy diagnostics were not available in N.Y.). Measurement of environmental toxins was done by both blood and urine testing. A urine DMSA challenge through Doctor’s Data was used to evaluate heavy metals (not just blood testing), since metals can leave the bloodstream and accumulate in tissues where they are no longer measurable, compartmentalizing in body tissues. DMSA effectively competes with tissue binding sites, releasing metals from sequestered sites, which then redistribute into the blood as a stable complex, and are then eliminated in the urine where they can be measured [109]. Patients were not tested with a DMSA challenge if there was evidence of significant sulfa sensitivity. Pesticide levels were measured by both blood (LabCorp) and urine analysis (PacTox Laboratories), and mold testing was done by urine analysis through the RealTime Laboratory in Texas, using liposomal glutathione (2 g) and sauna therapy prior to measurements [110]. G.I. evaluations included an upper endoscopy and colonoscopy if there was a history of GERD and/or colitis; H. pylori analysis was done by blood (and occasionally breath test, endoscopy); stool and microbiome analysis (bacteria, parasites, fungi) were done through both local laboratories and Genova/Metametrix. Liver function testing was done through local and national laboratories, with ultrasonography if NASH was suspected. Testing for Mast Cell Activation Disorder (MCAD) and food allergies included serum histamine, tryptase, chromogranin A, and IgE blood tests, respectively, (occasionally IgG4 delayed food allergy hypersensitivity testing was performed), when clinically indicated (MCAD can also be associated with POTS) [86]. Tilt table testing with or without small fiber biopsies and autonomic/electrodiagnostic testing (EMG) were done through private neurology and hospital settings (i.e., New York University). Sleep testing by polysomnography was done through Accusom (Novasom) home sleep testing (GlenBurnie, MD, USA) and in hospital settings with board certified pulmonary physicians (diplomates of the American Board of Sleep Medicine, Darien, IL, USA). A brief description of the tests and methods of evaluation for the 16 MSIDS variables appears on Table 1.

## 3. Results

### 3.1. MSIDS (Data Mining)

The 16-point Multiple Systemic Infectious Disease Syndrome (MSIDS) is “a symptom complex of Lyme disease and multiple associated tick-borne coinfections which encompasses not only infections with *B. burgdorferi*, the etiologic agent of Lyme disease, but also encompasses other bacterial, viral, parasitic, and fungal infections. This symptom complex also includes issues with immune dysfunction, inflammation, environmental toxicity, allergies, nutritional and enzyme deficiencies with functional medical abnormalities in biochemical pathways, mitochondrial dysfunction, neuropsychological issues, autonomic nervous system dysfunction, endocrine abnormalities, sleep disorders, GI abnormalities, with abnormalities of liver function, and issues with pain, drug use, and physical deconditioning” [111]. These abnormalities on the MSIDS model can be found in both those with Lyme disease, i.e., “Lyme-MSIDS”, and those with “non-Lyme MSIDS”, i.e., those individuals without tick-borne disease, where MSIDS variables may be contributing to the burden of chronic illness.

**Figure 1:**
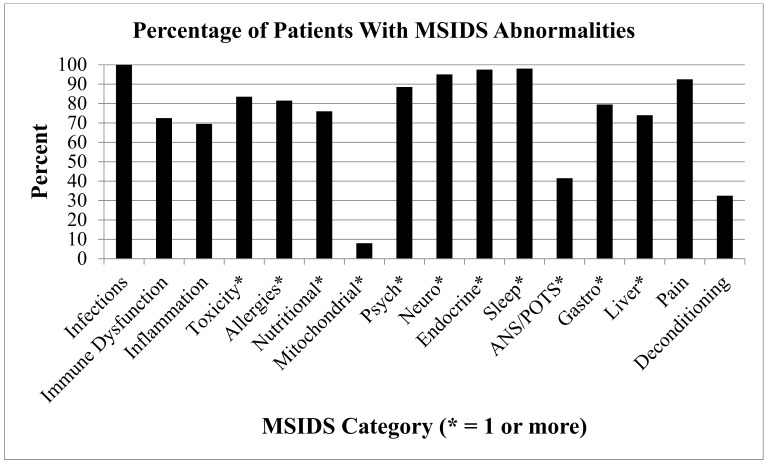
Percentage of patients with MSIDS abnormalities. Multiple overlapping abnormalities on the MSIDS map were associated with Lyme disease, contributing to ongoing symptomatology.

Since the MSIDS model posits that patients may remain ill with persistent Lyme symptoms not only because of ongoing infection(s), but also because of a complex of other overlapping medical problems, we closely examined each patient’s chart for evidence of these conditions and abnormalities. We found extensive evidence that patients did not have just Lyme disease alone. Figure 1 provides an overview of the percentage of patients experiencing each of the 16 points from the MSIDS map, data mined directly from the patient charts for the 200 participants in the study.

### 3.2. Patients Have More Than Lyme Disease: MSIDS Multifactorial Analysis

All our patients (100%) had evidence of exposure to one or more infections—all were being treated for Lyme disease as well as a range of coinfections (see Precision Medicine: Part 1). Babesiosis (*B. microti* and *B. duncani*) and *Bartonella* spp. (*B. henselae*) were the most commonly found coinfections. Among the 52% of patients with evidence of babesiosis by antibody titer and/or PCR/FISH, a significant percentage of *B. duncani* cases tested (28%) were found in the eastern seaboard. A small percentage of patients (2.5%) were found to have antibody titers for other *Bartonella* species, including *Bartonella quintana*. 

**Figure 2:**
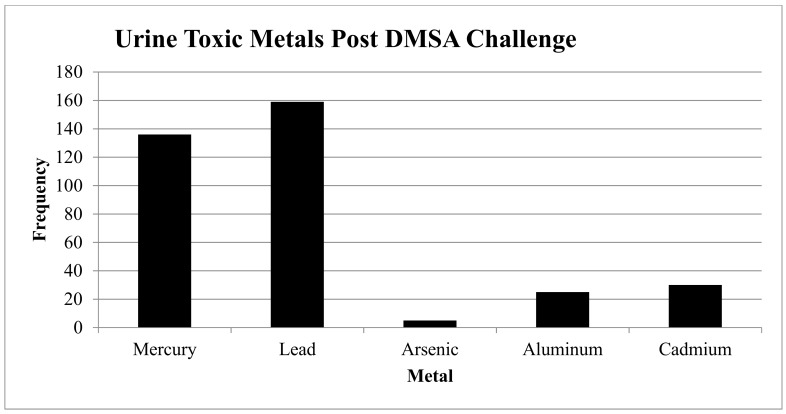
Urine toxic metals post dimercaptosuccinic acid (DMSA) challenge in 185 patients showed a high frequency of exposure to mercury and lead. Toxic metals were reported as micrograms/gram of creatinine to account for urine dilution variation. Ranges reported were within the reference range, elevated, and very elevated in comparison to a healthy population under non-challenge or non-provoked conditions.

Almost three quarters of patients (72.5%) had immune dysfunction (as measured by a positive ANA, RF with or without evidence of genetic HLA DR2, and DR4 markers with 85% having combined IgG subclass deficiencies). Several patients also had evidence of immune dysfunction based on the lack of increased antibody production in response to a pneumococcal vaccination. More than 69% of patients had evidence of inflammation (e.g., elevated ESR, CRP, TGFB1, C3a, C4a, and/or VEGF). Approximately 85% had positive tests for heavy metals, including elevated levels of lead, mercury, arsenic, cadmium, and aluminum (see Figure 2), with smaller numbers showing evidence of pesticide and mold exposure (not all patients were tested).

Large numbers of patients (81%) had positive allergy testing (food allergies [45%] and drug allergies [56%]), with more than three quarters (76%) having one or more nutritional and enzyme deficiencies (MTHFR mutations were present in more than half of the patients [52.5%]). We also found that most individuals suffered from sleep disorders (98%), endocrine abnormalities (97.5%), neurological dysfunction (95%), pain syndromes (92.5%), psychological issues (88.5%), and some form of gastrointestinal and liver dysfunction (79.5% and 74% respectively), with less than half of the individuals having evidence of varying degrees of POTS/dysautonomia (41.5%), deconditioning (32%), and mitochondrial dysfunction (7.5%). In addition to coinfection testing, which was positive in our patients (see Precision Medicine: Part 1), the following is a detailed analysis of the number of individuals with multiple overlapping factors on the MSIDS map associated with their illness:

- Immune Dysfunction (positive ANA, RF, HLADR2, HLADR4: 145 (72.5%) participants had immune dysfunction, 13.5% had elevated IgM antibodies, and up to 85% had some form of immune deficiency:
  ○ 20.6% had total IgG deficiency;
  ○ 19.3% had IgM deficiency; and
  ○ 15.9% had IgA deficiency.
  85.5% had combined IgG subclass deficiencies 1–4 (see Table 2).
- Inflammation (Elevated ESR, CRP, TGFB1, C3a, C4a, TNF, VEGF): 139 (69.5%) participants had markers of inflammation.
- Toxicity: See Figure 2.
  ○ Heavy Metals: 169/185 (84.5%) had one or more elevated heavy metals using a 6-h urine DMSA challenge:
    ▪ 159 (79.5%) had elevated lead levels (N = 73 were elevated, and N = 59 were very elevated);
    ▪ 136 (68%) had elevated mercury levels (N = 77 were elevated, and N = 59 were very elevated);
    ▪ 5 (2.5%) had elevated arsenic levels (N = 3 were elevated, and N = 2 were very elevated);
    ▪ 25 (12.5%) had elevated aluminum levels (N = 25 were elevated); and
    ▪ 30 (15%) had elevated cadmium levels (N = 26 were elevated, and N = 4 were very elevated).
  ○ Mold: 30/42 (71.4%) had one or more elevated mold levels:
    ▪ 13/25 (52%) had elevated aflatoxins;
    ▪ 18/26 (69%) had elevated ochratoxins;
    ▪ 20/26 (76.9%) had elevated trichothecenes;
    ▪ 17/17 (100%) had elevated gliotoxins; and
    ▪ 7/18 (38.9%) had other elevated mold (Stachybotrys exposure).
  ○ Pesticides: 5 (2.5%) tested positive for pesticides*.

*Not all patients were tested for mold or pesticides: Only those with a history of significant mold exposure and/or pesticide exposure were checked, especially if there was evidence of significant chemical sensitivity and/or Parkinson’s symptoms.

- Allergies: 163 (81.5%) of participants had allergies:
  ○ 90 (45%) had food allergies;
  ○ 43 (21.5%) had environmental allergies (e.g., seasonal allergies, allergy to animals, etc.);
  ○ 7 (3.5%) had high IgE levels;
  ○ 3 (1.5%) had high histamine levels (not all patients were tested for histamine sensitivity or Mast Cell Activation Disorder [MCAD]);
  ○ 112 (56%) had drug allergies; and
  ○ 12 (6%) had allergies categorized as “other”.
- Nutritional and Enzyme Deficiencies: 152 (76%) participants had one or more of these deficiencies. All patients were tested for mineral deficiencies, but only patients with poor nutritional intake were tested for amino acid and/or fatty acid deficiencies:
  ○ 5 (2.5%) had amino acid deficiencies;
  ○ 2 (1%) had fatty acid deficiencies;
  ○ 36 (18%) had iodine deficiencies;
  ○ 14 (7%) had copper deficiencies;
    ▪ 3 (1.5%) had deficiencies in serum copper;
    ▪ 6 (3%) had deficiencies in red blood cell [RBC] copper;
    ▪ 5 (2.5%) had deficiencies in plasma copper;
  ○ 31 (16%) had magnesium deficiencies;
    ▪ 6 (3%) had deficiencies in serum magnesium;
    ▪ 26 (13%) had deficiencies in RBC magnesium;
  ○ 36 (18%) had zinc deficiencies;
    ▪ 22 (11%) had deficiencies in serum zinc;
    ▪ 7 (3.5%) had deficiencies in RBC zinc;
    ▪ 7 (3.5%) had deficiencies in plasma zinc; and
  ○ 105 (52.5%) had MTHFR mutations.
- Mitochondrial Dysfunction (defined by those who had positive responses to the following mitochondrial support supplements: ATP fuel (NT Factors, i.e., glycosylated phospholipids), Coenzyme Q10 (CoQ10), acetyl-l-carnitine, d-ribose): 15 (7.5%) had mitochondrial dysfunction.
- Psychological issues: 177 (88.5%) participants self-reported having at least one psychological problem:
  ○ 154 (77%) had depression;
  ○ 134 (67%) had anxiety;
  ○ 4 (2%) had Obsessive Compulsive Disorder (OCD);
  ○ 11 (5.5%) had Post Traumatic Stress Disorder (PTSD); and
  ○ 9 (4.5%) had other psychological issues.
- Neurological Dysfunction: 190 (95%) had at least one of the following Neurological symptoms/disorders:
  ○ 188 (94%) had neuropathy;
  ○ 5 (2.5%) had Chronic Inflammatory Demyelinating Polyneuropathy (CIDP);
  ○ 3 (1.5%) had Multiple Sclerosis;
  ○ 2 (1%) had seizures; and
  ○ 2 (1%) had other neurological issues (e.g., Parkinson’s symptoms).
- Endocrine Abnormalities: 195 (97.5%) had at least one of the following endocrine abnormalities:
  ○ 121 (60.5%) had thyroid abnormalities;
  ○ 144 (72%) had adrenal abnormalities;
  ○ 82 (41%) had sex hormone abnormalities;
  ○ 136 (68%) had vitamin D deficiencies;
  ○ 3 (1.5%) had pregnenolone deficiencies; and
  ○ 74 (37%) had DHEA abnormalities.
- Sleep Disorders: 196 (98%) had at least one of the following sleep disorders:
  ○ 23 (11.5%) had Obstructive Sleep Apnea (OSA);
  ○ 1 (0.5%) had Restless Leg Syndrome (RLS);
  ○ 7 (3.5%) had Benign Prostatic Hyperplasia (BPH);
  ○ 4 (2%) were in menopause;
  ○ 2 (1%) had high adrenals;
  ○ 1 (0.5%) had medication induced sleep problems; and
  ○ 189 (94.5%) had other sleep problems, i.e., difficulties with insomnias, hypersomnias, circadian rhythm disorders (secondary to Lyme and tick-borne diseases).
- Autonomic Nervous System (ANS) Dysfunction/POTS: 83 (41.5%) had ANS dysfunction and/or POTS:
  ○ 23 (11.5%) had mild POTS (1–10 mm Hg drop in BP, and/or 1–10-point increase in heart rate after standing);
  ○ 41 (20.5%) had moderate POTS (11–29 mm drop in BP, and/or 11–29-point increase in heart rate after standing);
  ○ 9 (4.5%) had severe POTS (30+ increase in heart rate standing);
  ○ 19 (9.5%) had dysautonomia (e.g., gastroparesis, chronic constipation, bladder dysfunction, or dysfunction in temperature regulation); and
  ○ 2 (1%) had ‘other’ (tremors and/or discoloration hands/feet).
- Gastrointestinal Dysfunction: 159 (79.5%) had one or more of the following gastrointestinal disorders:
  ○ 10 (5%) had gluten sensitivity;
  ○ 10 (5%) had celiac disease;
  ○ 2 (1%) had colitis;
  ○ 43 (21.5%) had Candidiasis;
  ○ 15 (7.5%) had leaky gut;
  ○ 35 (17.5%) had parasites;
  ○ 17 (8.5%) had *H. Pylori* exposure;
  ○ 37 (18.5%) had gastroesophageal reflux disease (GERD);
  ○ 0% had a history of *C. Difficile* during treatment with dapsone; and
  ○ 83 (41.5%) had ‘other’ gastrointestinal dysfunction (Irritable Bowel Syndrome [IBS]).
- Elevated Liver Function Tests (LFTs): 148 (74%) had one or more of the following transient elevation in LFTs at some point during treatment
  ○ 90 (45%) had elevated AST;
  ○ 104 (52%) had elevated ALT;
  ○ 36 (18%) had alkaline phosphatase;
  ○ 47 (23.5%) had elevated T. Bilirubin; and
  ○ 5 (2.5%) had ‘other’ (low albumin).
- Pain Syndromes: 185 (92.5%) had migratory pain, which other research has demonstrated is one of the hallmark symptoms of active Lyme disease [39].
- Deconditioning: 64 (32%) were disabled and/or in physical therapy (PT).

## 4. Discussion

Results from Precision Medicine: Part 1 support and extend the findings of our published shorter term dapsone study in 100 patients on a dapsone protocol [106] for longer than three months. For the symptoms of Fatigue/Tiredness, Joint and/or Muscle Pain, Headache, Tingling/Numbness/Burning of the Extremities, Sleep Problems, Forgetfulness or Brain fog, Difficulty with Speech or Writing, and Day Sweats/Night Sweats/Flushing, 164 out of 200 patients (82%) had a statistically significant decrease of severity ratings of each symptom after treatment. Findings that differed from the 2016 study [106] include a significant decrease of symptom severity of headaches—this was not found previously. Although neuropathy (tingling/numbness/burning sensations) is a known potential side effect of dapsone [112], our study showed statistical improvement in this symptom when used in our three-drug antibiotic combination therapy. 

### 4.1. Numerous Health Issues Confound Full Recovery

Apart from noting the incidence of coinfections outlined in Precision Medicine: Part 1, markedly different from the Horowitz and Freeman 2016 [106] study was the inclusion of a medical chart review of all 200 participants for evidence of abnormalities on the 16-point MSIDS map. The MSIDS information notably suggests that these patients, all of whom had persistent ongoing symptomatology (prior to DDS combination therapy), remained symptomatic because apart from documented evidence of Lyme disease, they had numerous other health issues likely interfering with a full recovery. Some of the most important medical problems which needed to be addressed on the MSIDS model to ensure clinical improvement included adequate treatment of babesiosis and bartonellosis, decreasing inflammation (avoiding allergic/sensitive foods, getting adequate sleep), assisting detoxification pathways for severe Herxheimer reactions, addressing immune dysfunction and/or immune deficiency, treating POTS/dysautonomia, as well as addressing any associated hormonal and psychological dysfunction. These multi-faceted health challenges made these patients’ symptoms more difficult to treat. Chart review was also able to identify several effective combinations of antibiotics, as patients were frequently rotated through different combinations while on dapsone, as well as identify how multiple factors on the MSIDS map affected treatment outcomes.

In the three prior National Institutes of Health (NIH) randomized controlled trials for the treatment of Lyme disease [4,113,114], none of the above factors on the MSIDS map were considered. An analysis by Delong et al. [115] concluded that prior NIH double-blind treatment trials for Lyme disease produced mixed results. Although two of the three clinical NIH trials did show improvement in symptomatology, i.e., the Krupp trial showed improvements in fatigue, and the Fallon study showed improvement in encephalopathy with improved cognitive functioning, sustained clinical improvement was lacking. This is commonly observed in clinical practice once patients with chronic Lyme symptoms stop anti-infective therapy. 

Several explanations as to why the NIH studies did not find consistent clinical improvement [116] are that those studies did not incorporate recent up-to-date published scientific information on Borrelia and its ability to form biofilms [117] and “persister” bacteria [118,119,120], which were factors addressed in our study; these were inadequate treatment trials because sample sizes were extremely small, ranging from 37 to 78 patients. Sample sizes this small lack sufficient statistical power to measure clinically relevant improvement. Furthermore, ongoing coinfections, including *Babesia* spp. and *Bartonella* spp., may have been present in the participants, and these infections are treated differently than *Borrelia burgdorferi*; overlapping causes of inflammation were not adequately addressed in the NIH studies i.e., food allergies/sensitivities, leaky gut, imbalances of the microbiome, resistant insomnia, elevated levels of environmental toxins, including heavy metals and mold, with nutritional deficiencies in magnesium, copper, zinc, and/or iodine; nor were the downstream effects of inflammation addressed. These factors include, but are not limited to, endocrine disorders (low testosterone, low adrenal function), mitochondrial dysfunction, and POTS/dysautonomia. It is important to consider all these factors on the MSIDS model, since they can result in the same symptoms seen in chronic Lyme disease/PTLDS, including resistant fatigue, headaches, dizziness, anxiety and palpitations, neuropsychiatric symptoms with depression and anxiety, cognitive difficulties/problems with executive functioning, and insomnia. If simultaneous overlapping etiologies are present, which can increase an inflammatory process and cause similar symptoms, it will be difficult to differentiate the effectiveness of anti-infective therapy alone until all underlying etiologies increasing inflammation have been properly diagnosed and treated. 

### 4.2. Neurocognitive Deficits in PTLDS and Lyme-MSIDS

Multiple overlapping causes of inflammation may explain some of the differences in outcomes of studies comparing PTLDS patients to the larger group of patients with chronic Lyme disease. In a recent 2018 study on cognitive decline in 124 patients with PTLDS [26], 92% of patients had some level of cognitive difficulty, yet 50% had no statistically or clinically relevant cognitive decline, with only 26% showing significant cognitive decline on measures of memory and processing speed. In our study of 165 patients with “Lyme-MSIDS” who reported their symptoms before DDS and at least six months on DDS, almost 91% of patients self-reported some level of cognitive difficulty (similar numbers to the Touradji et al. study), but the group with moderate, moderately severe, or severe cognitive impairment (forgetfulness/brain fog) was three times higher at 78%. Speech and writing problems were present in more than three quarters of these patients (76.4%). These were also patients with evidence of inflammation (69.5%) and immune dysfunction (72%). Infections (including coinfections), heavy metal burdens (84.5%), nutritional deficiencies (76%), and sleep disorders (98%) as well as G.I. disorders (79.5%) can all increase inflammation and contribute in part to neurocognitive deficits. The role of infections, however, is striking. As we reported in Precision Medicine: Part 1, after six months of dapsone therapy, the group with the most significant cognitive deficits (moderate, moderately severe, and severe) statistically improved with DDS with *p* values < 0.001. The success of dapsone combination therapy using hydroxychloroquine, grapefruit seed extract, doxycycline, rifampin, DDS, and agents that disrupt the biofilm (stevia, oregano oil) [121] was probably due to several mechanisms of action. Clinically, this protocol has good penetration into the central nervous system (CNS), where it can exert its antibacterial effects (stopping RNA and protein production by bacteria); it works against a broad range of pathogens (i.e., *Borrelia* as well as multiple intracellular coinfections), with efficacy against different forms of *Borrelia*, including round body, stationary phase, and biofilm forms. Dapsone also has an anti-inflammatory effect, by converting myeloperoxidase (MPO) into its inactive compound II (ferryl) form [122]. Myeloperoxidase is an enzyme in neutrophils that results in the production of hypochlorous acid (HOCl), whose function is to help kill bacteria and other pathogens, although it can also cause inflammation/oxidative damage in tissues. Inflammation and inflammatory cytokine production has been linked to fatigue, pain (migratory pain was present in 92.5% of our patients, which is a hallmark symptom of active Lyme disease) [96], and neurocognitive deficits in large controlled studies [123].

### 4.3. The Role of Inflammation in Lyme Disease and MSIDS

How might inflammation affect patient outcomes and how might we control its effects? There are at least three major biochemical pathways that may be involved in the production of inflammatory cytokines, which include the NF-Kappa B [124] (NFK-B) and nitric oxide (NO) pathways [125,126], and biochemical reactions leading to production of advanced glycation end products (AGE’s) [127]. Multiple factors on the MSIDS map, including, but not limited to, multiple infections and environmental toxins, along with a high carbohydrate diet with hyperinsulinemia [128] and imbalances in the microbiome can create inflammation through stimulating these pathways, resulting in elevated levels of chemokines as well as cytokines, including IL-1, IL-6, TNF-α, and IL-17 [48,129,130,131,132,133]. These inflammatory cytokines are responsible for some of the disabling symptoms seen in Lyme disease, and their production along with the creation of free radicals/oxidative stress can damage cell membranes, mitochondria, and nerve cells. A potential solution is, therefore, to not only avoid simple carbohydrates and allergic/sensitive foods while treating the full range of infections and overlapping abnormalities on the 16 point MSIDS model increasing inflammation, but also to block NFK-B and NO (antioxidants, including alpha lipoic acid and glutathione, may be helpful) [134,135,136], while simultaneously increasing detoxification. Low dose naltrexone (LDN) has been one medication shown to also help in this regard, by decreasing microglial activation of the brain, and subsequently lowering CNS inflammatory cytokine production. LDN can be an effective treatment for autoimmune diseases, including Crohn’s disease and multiple sclerosis as well helping with fibromyalgia symptoms [137,138,139,140].

Lowering inflammation may also take place by stimulating the Nrf2 pathway in the cytoplasm of cells. Nrf2 acts as a sensor in the cytoplasm that regulates redox balance and the stress response: It is activated by oxidative stress [141]. Once activated, Nrf2 goes into the nucleus and binds to Antioxidant Response Element (ARE) genes. These are DNA binding sites that primarily activate phase II enzymes (with a minor effect on phase I) plus numerous other cytoprotective enzymes. ARE gene activation enhances detoxification, decreases inflammation, and inhibits cancer growth. This mechanism may explain many observed beneficial effects of detoxifying phytochemicals noted in the scientific literature, including a variety of substances which activate Nrf2: Sulforaphane [142](broccoli seed extract), resveratrol [143,144], green tea (epigallocatechin gallate [EGCG]), and curcumin [145]. Patients in our study were instructed to take antioxidants (NAC, alpha lipoic acid, glutathione), LDN (if not on narcotics for pain, since naltrexone blocks narcotic receptors), and Nrf2 activators during dapsone combination therapy. A more detailed statistical analysis in a larger cohort of patients will be necessary, however, to evaluate their relative efficacy of decreasing inflammation, compared to the effects of anti-infective therapy and correcting abnormalities on the 16-point MSIDS model.

### 4.4. Repairing Free Radical Damage: The 4 “R’s”: Replace, Repair, Rebalance, Re-inoculate the G.I. Microbiome

Once we addressed the multiple sources of inflammation and used drainage and detoxification support with glutathione (GSH), then repairing the damage to the body may be helpful. This can be summed up by “the 4 R’s”: Replace (hormones), Repair (mitochondria) [61,146], Rebalance (ANS) [82,147,148], and Re-inoculate (G.I. bacteria) [149]. Almost all (97.5%) of our patients had endocrine abnormalities; 7.5% of our patients had mitochondrial dysfunction; 41.5% had some level of POTS/dysautonomia, and 79.5% had some form of gastrointestinal dysfunction. Retrospective chart review showed hormone balancing (adrenals, sex hormones, thyroid) and addressing POTS/dysautonomia to be key factors in those with resistant fatigue and cognitive dysfunction. 

Protecting and inoculating the right types of bacteria (and yeast, i.e., *Saccharomyces boulardii*) was also important. The type of bacteria in the microbiome of our gut can have different effects on inflammatory cytokine production. Some of these bacteria, such as *Prevotella* and *Clostridium* species, have recently been reported to be associated with inflammation and diverse disease manifestations, including rheumatoid arthritis [150] and multiple sclerosis (MS) [151,152,153,154]. All our patients were on probiotics to support a healthy microbiome throughout the G.I. tract (*Lactobacillus* strains are most active in the small intestine; *Bifidobacterium* strains work best in the large intestine). These strains were chosen as they have been shown in double blind studies to help with functional bowel disorders [155] (*L. acidophilus* NCFM, *bifidobacterium Bi-07*); possess anti-inflammatory and immune enhancing properties [153,156,157] (*bifidobacterium Bl-04* increases IL-10, and *B. lactis* can enhance immunity in the elderly); while helping to prevent *C. difficile*. According to the CDC, nearly half a million Americans suffered from *Clostridium difficile* infections in a single year [158] and more than 100,000 of these infections developed among residents of U.S. nursing homes [158]. Some of these may have been preventable with regular use of targeted probiotics, such as *Saccharomyces boulardii*. Double-blind studies have shown targeted probiotics to help with functional bowel disorders, and large-scale clinical studies need to be performed to evaluate their efficacy in reducing associated morbidity.

It is important to identify potential multifactorial causes of chronic diseases, since health care costs are rising, with a recent health care survey reporting that almost half of all Americans suffer from at least one of 10 chronic conditions [159]. Autoimmunity can result from antibodies produced against *Borrelia* that cross react with our own tissue antigens (molecular mimicry), while bacteria activate Toll-like receptor 2 (TLR-2), [160,161] furthering increases in the pro-inflammatory cytokines, TNF-alpha and IL-17. Similarly, mercury (Hg), bisphenol A (BPA), asbestos, and small particle pollution have now been published in the medical literature as potential factors increasing autoimmune reactions [37,51,101,102]. Over 84% of patients had evidence of exposure to at least one heavy metal, with 68% had evidence of mercury exposure; 100%, 46.5%, and 82% of patients had evidence of exposure to *Borrelia*, *Bartonella*, and *Mycoplasma* spp., respectively; and 72.5% of our patients in this study had evidence of immune dysfunction, including production of positive antinuclear antibodies and rheumatoid factors. Although ANA’s and rheumatoid factors can be seen in autoimmune diseases, including systemic lupus erythematosus (SLE) and rheumatoid arthritis, these represent non-specific markers of inflammation in our study (only three patients were positive for dsDNA and/or CCP antibodies, markers of true lupus and rheumatoid arthritis). There was also evidence of elevated inflammatory markers, including elevated sedimentation rates (ESR), C-reactive protein (CRP), and C3a/C4a, in most of our patients, although the latter can be seen with both Lyme disease [162] and toxic mold exposure [163]. Forty five percent had evidence of food allergies (not everyone was checked for evidence of leaky gut by breath test analysis), and Vitamin D deficiency was noted in 68% of patients, with several patients having elevated 1,25 dihydroxyvitamin D/25-OH vitamin D ratios (>2:1 ratio), suggestive of an active intracellular infection increasing inflammation [164]. Interestingly, the three patients identified with elevated 1,25 dihydroxyvitamin D/25 OH vitamin D ratios also had evidence of immune deficiency (decreased immunoglobulin levels, decreased antibody production to a pneumococcal challenge, and/or a genetic predisposition). In total, 139 (69.5%) participants had markers of inflammation. These biomarkers of inflammation, including the recently identified chemokine, CCL-19 [133], can be followed during the course of treatment to help confirm clinical improvement in symptoms. 

**Inflammation in Other Chronic Conditions:** Lyme disease patients are known to have increased levels of IL-1, IL-6, and TNF-α, also found in chronic fatiguing, musculoskeletal illnesses, and other neurodegenerative disorders. These pro-inflammatory cytokines increase fatigue and pain as well as peripheral nervous system (PNS) and central nervous system (CNS) neurological symptoms [165,166,167]. They can be produced by not only infections [47], but environmental toxins. [125,168] In recent years “more than 100,000 new chemicals have been used in common consumer products and are released into the everyday environment” [169]. According to a 2018 report of the US population, 12.8% now report medically diagnosed multiple chemical sensitivity (MCS) and 25.9% report chemical sensitivity [170]. Treatment of chemical exposure/sensitivity, which may result in some of the same increased inflammatory cytokines seen in Lyme disease, requires a different therapeutic approach. Chemical sensitivity is best addressed by decreasing our total exposure and toxic load (i.e., reducing the body burden through far infrared saunas, chelation, and mold removal using phosphatidylcholine and glutathione) [171], while supporting the detoxification pathways (phase I and phase II liver support, methylation support, toxin binders). Our study and chart review showed that evaluating patients for associated environmental toxicity and using detoxification with glutathione (GSH) is clinically useful, especially during Herxheimer reactions. Glutathione modulates inflammation [172,173]. A 2018 PNAS report found that intracellular GSH production was increased up to 10-fold during an infection with *Borrelia burgdorferi*, and infection was a key factor in inflammation/cytokine production [174]. Glutathione is also a key factor in helping to detoxify environmental chemicals.

Environmental toxins are known to be detrimental to our health, but what is their potential role in patients with Lyme disease and how significant is our daily exposure to a broad range of pollutants? Apart from evaluating patients for exposure to heavy metals and mold toxins, which were present, respectively, in 84.5% and 71.4% of those tested, we only evaluated five individuals for pesticide exposure, some of whom had Parkinsonian symptoms. Other published scientific research suggests our exposure to a broad range of toxins, including pesticides, to be much greater. The CDC performed a 6.5-million-dollar study in 2003, evaluating 2500 patients for environmental toxins [175]. They found a total of 116 different pollutants (13 heavy metals, 14 combustion byproducts, and 10 pesticides). One of those toxins, trichloroethylene (TCE), caused a leukemia outbreak in Woburn, Massachusetts. TCE can also cause learning disabilities and paresthesias, as can exposure to mercury and lead [38]. These are some of the same symptoms we see in neurological Lyme disease with or without coinfections. Some patients who undergo detoxification, such as using Far infra-red saunas, with or without oral (or IV) liposomal glutathione, notice clinical improvement in what was perceived to only be Lyme related symptoms. Herxheimer reactions also oftentimes improves with alkalinizing the body [176] (decreasing acidic byproducts) and using liposomal glutathione to support detoxification. How can we potentially use this information to inform future clinical studies and research and improve patient care?

### 4.5. Important MSIDS Variables Determining Treatment Outcomes

The key points which emerged from a detailed data mining and review of these 200 patients charts with PTLDS/chronic Lyme disease were that a combination of factors on the 16-point MSIDS map needed to be addressed to see maximum improvement in patient symptoms. This usually involved the successful treatment of: 

1. **Multiple intracellular infections:** Including *Borrelia*, *Bartonella*, *Chlamydia*, and *Mycoplasma* [32], using a triple intracellular antibiotic combination therapy (doxycycline, rifampin, and dapsone) along with agents that disrupt biofilm (i.e., Stevia [177], oregano oil [178]). Although *Borrelia* has been reported to exist in the intracellular compartment [179,180,181,182], the importance of addressing *Borrelia* and associated intracellular infections in clinical studies of patients with chronic Lyme symptoms has been a subject of ongoing debate among clinicians and scientists. Although concurrent infection with more than one agent is already known to complicate management of patients [183,184,185], our new study on dapsone combination therapy implies a significant role of intracellular bacteria in those suffering from symptoms of chronic Lyme disease/PTLDS. Our 2016 and new study imply that there may be multiple intracellular bacterial infections present in a subset of Lyme patients with persistent symptoms, some of whom have autoimmune manifestations, and a broad screening approach is necessary, using multiple testing strategies over time. 

2. **Parasitic infections:** *Babesia* oftentimes required rotations of antimalarial medication and herbal therapies, due to the ability of *Babesia microti* to persist after standard treatments with drugs, like atovaquone and azithromycin [186,187,188]. Patients usually reported feeling better once Babesiosis was adequately treated. *Babesia* infection is also known to interfere with the clearance of other parasites. Addressing these other parasites and co-morbid conditions oftentimes led to clinical improvement. Although dapsone combination therapy was effective in reducing symptoms of babesiosis (decreased fevers, sweats, chills, flushing) as it has an antimalarial effect, further research is needed to identify more effective treatments for babesiosis, since several patients had evidence of persistent symptoms and positive *Babesia* PCR’s or RNA (FISH) testing despite standard therapies. The role of associated parasitic infections and their interactions with *Babesia* parasites and host immunity needs further study.

3. **Sleep disorders:** These needed to be addressed by treating Lyme and associated coinfections, as well as ruling out other causes of sleep related problems (such as OSA, RLS, BPH, depression, anxiety, long acting stimulant and/or caffeine use). Treatment of insomnia using various medications (i.e., trazadone, tiagabine, mirtazapine, cyclobenzaprine, and pregabalin) and herbal therapies which support sleep and the circadian rhythm (i.e., valerian root and melatonin, which also lowers IL-17) were often needed [189]. Chart review indicated that balancing hormones (phosphatidylserine can be used to lower high adrenal function at night) [190] while stimulating GABA receptors (using GABA L-theanine) also were occasionally helpful in getting the patient to sleep. 

4. **Hormonal dysregulation:** Low hormones (adrenal, sex hormones, thyroid) needed to be corrected to see maximum improvement in fatigue, libido, and cognitive and weight challenges in certain patients [191,192]. Forty-one percent of patients had hormonal dysregulation, and low testosterone (low T) was occasionally seen in young men in their 20’s, 30’s, and 40’s with low libido. One individual (39 years old) with Lyme disease had a testosterone level of 138 (normal range 250–1100 ng/dl, Quest Diagnostics), whose testosterone increased to 356 with clomiphene (Clomid) and anastrozole (Arimidex). The use of clomiphene [193] and anastrozole (an aromatase inhibitor) [194] in men has been shown to improve testosterone/estrogen ratios without additional use of testosterone replacement therapy, which has its limitations and potential side effects. 

5. **Autonomic Nervous System dysregulation:** POTS/dysautonomia needed to be adequately addressed in a subpopulation of our patients if there was resistant fatigue, dizziness with changing position, presyncopal or syncopal episodes, unexplained palpitations and anxiety, as well as resistant cognitive symptoms [82]. The most commonly used therapies resulting in clinical improvement of dysautonomia was a combination of salt, increased fluids, and/or medication, including fludrocortisone, midodrine [195], and/or droxidopa [196], for blood pressure support, and Beta blockers (metoprolol XL) for control of palpitations. Further data mining analysis will be needed to determine the most effective combination of these medications, as patients oftentimes required more than one medication to improve symptomatology. In total, 41.5% of patients had evidence of ANS dysfunction or POTS as per clinical definitions [86]. Dysautonomia has also been reported in M.E./Chronic Fatigue Syndrome [197].

6. **Immune dysfunction/immune deficiency:** This occasionally required subcutaneous or intravenous immunoglobulin therapy (IVIG), as immunoglobulins are necessary to fight infections (CVID), modulate immunity [198], heal (small fiber) neuropathy, POTS, and address autoimmune encephalopathy [199]. Improvement of CVID using embryonic stem cell therapy in Lyme disease may be an option in resistant disease [200]. More than 72% of participants had immune dysfunction. Total IgG deficiency was found in 20.6%; 19.3% had IgM deficiency (13.5% had elevated IgM antibodies, not in the range of Waldenstroms macroglobulinemia); 15.9% had IgA deficiency (which can increase the incidence of food allergies), while more than 85% had combined IgG subclass deficiencies, 1–4 (see Table 1). The most frequent subclass deficiencies seen were IgG subclasses 1 and 3, associated with phagocytosis and antibody-dependent cellular and complement-dependent cytotoxicity. Some patients with normal or low normal immunoglobulin levels were also unable to mount an adequate antibody response to a pneumococcal challenge. Immune dysfunction and immune deficiency were therefore oftentimes associated with Lyme disease in our study, but the difficulty in determining the etiology and true prevalence was that there were too many potential overlapping variables (coinfections, heavy metals, mold toxins), which can all affect immunity (all patients tested for mold had evidence of gliotoxins, which are immunosuppressive). There is also the possibility that the patients who enrolled in the dapsone trial may have been sicker and more resistant to other therapies secondary to an immune deficiency. 

Another possible explanation comes from a study done by Nicole Baumgarth and researchers from the University of California at Davis. They found that infection with *Borrelia burgdorferi* in mice targets lymph nodes and production of IgG antibodies by affecting germinal centers, structures that are needed for the generation of highly functional and long-lived antibody responses. *Borrelia* subverted a B cell response in that study (B cells produce antibodies to fight infection), and instead, caused T cell independence, leading to an IgM skewed profile [201]. In our precision medicine study, we saw many more CDC IgM positive Western blots in patients with chronic persistent Lyme symptoms (N = 90, 45%), as opposed to IgG positive Western blots (N = 23, 11.5%), and 11.5% had increased IgM antibody production (N = 23). Thirty-three patients out of 171 tested (16.5%) also had low IgM antibodies. The incidence of low IgM antibodies in our study could represent the production of Lyme antibody-antigen complexes [202], and be a potential surrogate immune marker for active Lyme disease. Future studies in a larger cohort of patients will need to be performed to confirm this theory, although sequestration of antibody to *Borrelia burgdorferi* in immune complexes in the blood and spinal fluid in seronegative and neurologic Lyme disease have been reported [202,203] (one of our patients in the study who was initially negative on Lyme testing on a lumbar puncture was later found to be Lyme antibody-antigen complex positive in the spinal fluid in research assays performed at the State University of New York at Stony Brook). Over 19% of patients who failed DDS combination therapy (N = 36) had low IgG levels (14 had CVID) and 22.2% had low IgG subclass 1 (N = 8), while 36% (N = 13) had low IgG subclass 3. Immunoglobulin G and subclass deficiencies associated with Lyme and associated co-infections might therefore have contributed to DDS failures. A 2018 study [204] showed that robust B cell responses predict rapid resolution of Lyme disease. These immunological abnormalities found in our study parallel those discovered in mice exposed to *Borrelia burgdorferi* and may therefore explain, in part, persistent symptomatology in those with chronic Lyme disease/PTLDS. 

7. **Food allergies/sensitivities:** Avoidance of allergic and sensitive foods, which are known to increase the same inflammatory cytokines in Lyme disease, were helpful in our cohort of patients, and 45% of participants had evidence of food allergies, with 15.9% showing evidence of an IgA deficiency. Patients were instructed to stay on a low carbohydrate, Mediterranean/Paleo style diet, eating small frequent meals to avoid blood sugar swings (reactive hypoglycemia), Candida overgrowth in the G.I. tract, and further inflammatory reactions. Hypoglycemia may cause some of the same symptoms frequently seen in Lyme disease (fatigue, headaches, palpitations, mood swings, cognitive difficulties) [205], and both Paleolithic and Mediterranean diet pattern scores have been shown to be inversely associated with biomarkers of inflammation and oxidative balance in adults [206]. Mitochondrial damage can also be a by-product of excess sugar [146], resulting in metabolic syndrome and hyperinsulinemia [207] with insulin resistance, as glycation results in advanced glycation end products (AGEs) [127]. These can bind to receptors for advanced glycation end products (RAGEs), increasing inflammation [128,192] and cytokine production, which have been associated with nerve complications (neuropathy) [208] and neurological diseases (including Alzheimer’s) [209]. 

8. **Mitochondrial support:** In our study only 7.5% of patients reported an improvement in symptoms with the use of NT Factors, CoQ10, and acetyl-l-carnitine, and prior scientific research has shown that up to one third of patients with chronic Lyme symptoms respond to lipid replacement therapy [61,146]. Overlapping etiologies increasing inflammation that may not yet have been adequately addressed at that point in time during therapy could account for some of the discrepancies. Our definition of mitochondrial dysfunction may also have been too strict (response to mitochondrial support), since fatigue, muscle pain, nerve pain, and cognitive difficulties improved with dapsone combination therapy, and these symptoms have all been shown to be associated with mitochondrial dysfunction. Mitochondrial membranes are particularly vulnerable to free radical/oxidative stress, since they are not protected, as is DNA surrounded by histones [210]. Future studies should consider directly measuring markers of oxidative stress, such as lipid peroxides [211], Thiobarbituric acid reactive substances (TBARS), 8-OH d guanine [212], and protein carbonyls, since oxidative stress from infections and environmental chemicals has been shown to activate multiple signaling pathways, including NFK-B, leading to elevated cytokine production and inflammation [213]. One of our patients with chronic resistant symptoms and multiple infections had elevated levels of lipid peroxides (patients were not consistently measured for oxidative stress markers). Another patient with resistant neuropathy and autonomic nervous system dysfunction had a negative genetic workup to rule out inherited mitochondrial DNA (mtDNA) mutations [214]. A third patient was found to have mitochondrial dysfunction through the Cleveland Clinic with a low CoQ10 ratio, who improved with nutritional supplementation. Checking levels of amino acids, organic acids, ammonia, lactate/pyruvate ratios, creatinine phosphokinase (CPK), free/total carnitine ratios, and CoQ10 and tiglyglycine levels (a marker for mitochondrial respiratory chain disorders) can also be useful to evaluate mitochondrial function [215,216].

9. **Detoxification/Herxheimer support:** Glutathione and other detoxification support (NAC, alpha lipoic acid, methylation support, binders, drainage) were often helpful in reducing symptomatology, but due to challenges of identifying a broader range of toxins (expense, necessity of multiple blood draws and/or fat biopsies), lack of consistency in testing and treatment protocols, and because simultaneous overlapping inflammatory factors on the MSIDS model were often present, requiring different, individualized therapies, it was difficult to identify the exact role of these toxins and the effect of detoxification on environmental chemicals. Our conclusions are also limited in the absence of data on a healthy population tested under provoked conditions. More than 84% of our patients had evidence of exposure to heavy metals, including mercury, lead, and arsenic, and 71.4% had exposure to at least one mold toxin (not every individual was tested). The need to address mineral deficiencies and support detoxification pathways becomes particularly relevant in the setting of daily exposure to multiple environmental toxins, as 76% of our patients in the study had nutritional and enzyme deficiencies. 

10. **Mineral Replacement:** Seven percent of patients had evidence of copper deficiency. Copper is necessary to produce the enzyme, superoxide dismutase (SOD) [217], which helps control oxidative stress and mitigates subsequent free radical damage to cells. Sixteen percent of patients had evidence of magnesium deficiency. Magnesium is necessary in over 300 enzymes in the body, some of which are necessary for detoxification [218]. Testing for red blood cell levels of magnesium (and other minerals) was important in this context, since 13% of patients showed evidence of RBC magnesium deficiency, which would otherwise not have been found on routine serum analysis. Eighteen percent of patients were iodine deficient. Iodine helps support proper thyroid function [219]. Finally, 18% of patients were deficient in zinc, by both serum, plasma, and RBC analysis. Zinc is necessary for not only decreasing the production of inflammatory cytokines [220], but also acts as a cofactor in the enzyme, alcohol dehydrogenase [221]. Zinc deficiency may lead to aldehydes being formed from alcohol groups, and “oxidative stress, lipid peroxidation, hyperglycemia-induced glycations, and environmental exposures increase the cellular concentrations of aldehydes” [222], potentially increasing levels of chloral hydrate in the body. Chloral hydrate is an FDA-approved soporific agent used for resistant insomnia. Lyme patients with elevated levels of quinolinic acid [223], a byproduct of the L-tryptophan pathway, combined with ammonia (due to methylation defects, mitochondrial urea/organic acid disorders, and/or an imbalance of ammonia producing bacteria in the gut) along with chloral hydrate are all potential factors that might result in a worsening of neurocognitive symptoms [224], apart from exposure to Lyme and associated coinfections. These factors need to be evaluated in future clinical studies. 

Several patients with cognitive dysfunction and no evidence of severe liver disease (i.e., cirrhosis) had high ammonia levels. Ammonia, a byproduct of the metabolism of nitrogen-containing compounds, is neurotoxic at elevated concentrations [225] and the liver clears most portal vein ammonia, converting it into glutamine and urea. Glutamine is metabolized in mitochondria, yielding glutamate and ammonia, and ammonia can evoke oxidative/nitrosative stress, mitochondrial abnormalities, and astrocyte swelling, which is a major component of brain edema [226]. Ammonia levels can be decreased by reducing intestinal production (lactulose, rifaximin, diet), L-ornithine, prebiotic, and probiotic supplementation [227], as well as by supplementation with zinc and L-carnosine [228]. Zinc deficiency was found in 18% of patients, which could have impacted cytokine and ammonia production, as well as the integrity of the blood-brain barrier. Zinc deficiency superimposed on oxidative stress may predispose the brain to damage mediated though blood-brain barrier disruption [229]. Future studies will need to evaluate these mineral deficiencies and their role in chronic illness. 

Taking a comprehensive multifactorial approach to address all abnormalities on the MSIDS map, especially those resulting in inflammation through various pathways and leading to downstream effects on hormones and the autonomic and central nervous system, as well as sleep, was especially important in those patients with chronic resistant symptomatology despite standard therapies.

Almost three quarters (72.5%) of our patients in this study had evidence of immune dysregulation, and some of these had associated immune deficiency, including CVID (7%) and/or selective subclass deficiencies (85.5%). IgG1 and IgG3 subclasses contain proteins, which are usually mobilized against toxins (i.e., diptheria, tetanus) and viral proteins, through direct (antigen binding) or by mediating indirect effector functions, such as antibody-dependent cellular cytotoxicity (ADCC), antibody-dependent cellular phagocytosis (ADCP), and complement-dependent cytotoxicity (CDC) [230]. IgG2 antibodies are used predominantly against bacteria with capsule polysaccharides (S. pneumonia, H. influenzae), and while the role of IgG4 is still being studied, decreased levels can be associated with IgA deficiency (along with IgG2 deficiency) and a Th-1 activation in Lyme borreliosis, while elevated levels have been associated with systemic fibro-inflammatory disorders of unknown origin [231]. 

Prior examination of the IgG subclass distribution in Lyme borreliosis showed the predominating subclasses in both serum and CSF were IgG1 and IgG3 [232]. The T helper type 1 (Th1) IFN-gamma-predominated immune response seen in Lyme borreliosis results in the production of IgG1 and IgG3 subclasses that are complement activating and opsonizing. It has been hypothesized that increased levels of these two subclasses early in disease might contribute to recovery and counteract the development of chronicity. The IgG1 and IgG 3 subclass deficiencies noted in our patient population could therefore represent active viral (herpes viruses, i.e., EBV, CMV, HHV6) and/or intracellular bacterial infections, including *Anaplasma*, *Borrelia*, *Bartonella*, *Mycoplasma*, *Chlamydia*, *F. tularensis*, and *Brucella* (apart from toxin production), since these “mediated effector functions are especially important against infectious diseases where cellular and complement mediated responses are important for efficient pathogen clearance” [230,233]. These subclass deficiencies may also represent associated immune markers of active infection in Lyme and associated coinfections (along with low IgM antibodies, see Table 1), and need to be studied further in a larger cohort of well-defined patients. 

The relative prevalence of CVID in the general population (1:25,000 to 1:50,000) is usually much lower than those seen in our study (N = 14 [7%], i.e., approximately 1:14), resulting in acute and chronic infections, inflammatory, and autoimmune diseases [234]. Although genetic mutations have been identified, including those causing IgA and IgM antibody deficiency with autoimmune phenomenon [235], the precise etiology is usually unknown [236]. Lyme disease has been associated with cytopathic killing of lymphocytes [237] and immunodeficiency syndromes, as long-lived humoral immunity and immunoglobulin production can be suppressed after an infection with *Borrelia burgdorferi* [238]. Coinfections, like *Mycoplasma* [34] and *Bartonella* [239,240], as well as environmental toxins have also been associated with immunological dysfunction. *Mycoplasmas* can interact with B cells [33], affecting antibody production, and mercury [50], along with other environmental toxins [51] (including gliotoxins, which have been shown to be immunosuppressive), have been linked to immune dysfunction and a worldwide increase in autoimmune disease [37,241]. 

Although immune deficiency is known to increase the risk of infection, is infection increasing the risk of immune deficiency? Do chronic viral infections found in our study, like HHV6, play a role in Lyme and associated coinfections? HHV-6 is ubiquitous, can establish a lifelong, latent infection in its host, and is known to be a major cause of opportunistic viral infections in immunosuppressed individuals [242]. Certain flaviruses, like West Nile virus, found in our study (N = 13, 6.5%) have also been shown to be persistent [243]. Is the ability to clear multiple infections affected by heavy metals, like mercury, which have been associated with autoimmune phenomenon? One of the mold toxins found in our study, gliotoxins, is known to be immunosuppressive. Do mold and heavy metals, when combined with infections that are immunosuppressive, lead to more severe and resistant illness? Do these toxins increase the severity and duration of fatigue, pain, and neuropsychiatric symptoms when combined with Lyme and associated coinfections? Seventy-seven percent of patients reported depression in our study, and *Borrelia burgdorferi* and associated diseases have been associated with immune mediated and metabolic changes increasing the risk of suicide [40]. Since Lyme disease and environmental toxins both increase inflammatory cytokines, worsening mood disorders, does this increase risk? These are questions that will need to be answered in future scientific trials using a broad data mining approach. 

One important characteristic of our study that limits our ability to answer the above questions is that our patient population could represent a subset of the sickest patients with PTLDS/chronic Lyme disease, and not necessarily represent the broader population with chronic “unexplained” symptoms. Although our patient population did come from broad geographical areas across the US, we propose doing a national multicenter trial with a large cohort of patients who could be assessed using the 16-point MSIDS model, evaluating patient reported symptom severity and changes in MSIDS variables before and after treatment of each abnormality on the map. As previous research has demonstrated, self-reported symptoms can be reliable predictors of health outcomes [244]. In the future, we could measure cytokine levels, chemokine levels, and other inflammatory markers pre- and post-therapy to further evaluate the efficacy of DDS CT and treating multiple abnormalities on the 16-point MSIDS model, and include a healthy cohort for a detailed comparison chart review of all 16 factors. Our study showed improvement in the primary symptoms of Lyme disease and babesiosis, using a novel “persister” drug regimen. Multiple factors on the MSIDS map were also found to interfere and contribute to ongoing resistant symptomatology. Most of our patients improved when all sources of inflammation were discovered and adequately treated, along with treating the four “R”s. This needs to be confirmed in larger clinical studies. 

### 4.6. Healthcare Opportunities Going Forward

#### The Role of Biofilms and “Persisters” in Chronic Disease

Biofilms have recently been implicated as a possible factor in the pathogenesis of Alzheimer’s disease [245], just as they have been in Lyme disease, and play a role in some resistant infections (including, but not limited to, chronic otitis media, rhinosinusitis, implant infections with *Staphylococcus*/*Streptococcus*, urinary tract infections with *Pseudomonas*, *Candida* and *pneumocystis*) [246,247,248,249,250,251]. “Persisters are a specific subpopulation of bacterial cells that have acquired temporary antibiotic-resistant phenotypes” [252] and some are produced in higher numbers in colony–biofilm culture than in the usual liquid culture. Miyaue et al. [252] reported that “persisters can be maintained in higher numbers … even after complete withdrawal from the colony–biofilm culture. This suggests the presence of a long-retention effect, or “memory effect”, in the persister cell …” “… Increases in persisters during colony–biofilm culture and their memory effects are common, to a greater or lesser degree, in other bacterial species” [252]. *Borrelia burgdorferi* can form both biofilms [177] and persister cells [120], and the success of DDS combination therapy with agents that disrupt biofilms (Stevia, oregano oil) may be due to its effect against these different forms.

Patients infected with *Borrelia burgdorferi* and associated coinfections are often much sicker than patients with Lyme disease alone [19,65]. They can often be resistant to standard therapies (the role of biofilms and persisters in tick-borne disease still needs to be established in well controlled studies), but many in our study also had evidence of other medical problems, which might account for their ongoing symptoms. These health issues included immune dysfunction/immune deficiency, inflammation, environmental toxin exposure with detoxification problems, gastrointestinal problems, allergies, and nutritional deficiencies, as well as sleep, hormone, and autonomic nervous system dysregulation. Therefore, as other authors have suggested, we propose that patients with “chronic Lyme disease” no longer be referred to using this nomenclature, which can be confusing [253]. We suggest that patients now be considered to have “Lyme-MSIDS”, and believe that this term best describes the multiple biologic and biochemical abnormalities that can be present after an infection with *Borrelia burgdorferi* (whose etiologies go beyond tick-borne disease), causing chronic illness. Each patient is unique, and each treatment approach must be individualized (although certain guidelines for differential diagnosis and treatment remain in place based on peer reviewed literature and physician experience). The MSIDS model helps provide a framework for diagnosing and treating complex chronic Lyme disease patients that have a multiplicity of symptoms, along with a map of up to 16 potential factors that may need to be addressed. These factors can keep a patient chronically ill, but these abnormalities are not regularly accounted for in routine standards of patient care. Establishing a uniform definition of chronic Lyme disease will facilitate diagnostic and treatment decisions and allow comparison among varied cohorts of patients. 

## 5. Conclusions

The rising numbers of individuals suffering with Lyme disease and other long-term disabling illnesses alerts us to a necessary shift in the paradigm for the diagnosis and treatment of chronic disease [39]. An integrated, interdisciplinary systems-biology approach described in this study may help us to better understand Lyme and associated diseases. Based on published scientific data, we need to examine the role of multiple infections and environmental toxicants in neurodegenerative diseases, along with other factors on the MSIDS map that increase inflammation and cause downstream effects, including immune suppression. Some of these infections, like *Anaplasma*, *Babesia*, *Bartonella*, *Rickettsia* (Rocky Mountain spotted fever), and Relapsing Fever *borrelia*, now are present in the blood supply. *Borrelia*, *Babesia*, and *Bartonella* can be transmitted from a mother to her developing fetus, resulting in potential perinatal morbidity and mortality. *Rickettsia rickettsii* (Rocky Mountain spotted fever) can cause severe illness and death in children and adults if not treated within the first few days of illness [254]. Chronic tick-borne diseases can be both emotionally and financially devastating to individuals and their families and to the productivity of our country. Obstacles to the diagnosis and treatment of Lyme disease in the U.S. have also been associated with a “detrimental impact…on the ability to work and fulfill caregiving roles” [255]. 

A precision medicine focus and paradigm shift in health care is desperately needed. In attempting to fully understand the etiology(ies) of chronic Lyme disease/PTLDS, retrospective chart review and data mining research might help us to better understand etiologies and effective treatments for a broad range of other chronic diseases. Computer assisted, data mining from large cohorts of patients from multiple medical centers using a focused, personalized, precision medical perspective, like the MSIDS model, would allow us to examine the complexities of overlapping causes of inflammation in patients with ongoing suffering from Lyme and associated tick-borne disorders. 

## Figures and Tables

**Table 1:** MSIDS variables: Tests/method of evaluation.

**1. Infections**	Laboratory tests for the presence of *Borrelia* spp., *Babesia* spp., *Bartonella* spp., *Rickettsia* spp., etc.
**2. Immune Dysfunction**	Laboratory tests for autoimmune markers (ANA, RF), HLA status, immunoglobulin levels, and subclasses
**3. Inflammation**	Laboratory tests for markers of inflammation, i.e., ESR, CRP, TGFB1, C3a, C4a, and/or VEGF
**4. Toxicity**	Laboratory tests for heavy metals, mold toxins, pesticides, etc.
**5. Allergies**	Laboratory tests for IgE levels, food and environmental allergies, histamine, etc.
**6. Nutritional and Enzyme Deficiencies**	Laboratory tests for amino acids, fatty acids, mineral levels (serum, plasma, red blood cell)
**7. Mitochondrial Dysfunction**	Clinical evaluation of response to mitochondrial support (NT Factors, CoQ10, l-carnitine), evaluation of mtDNA mutations, etc.
**8. Psychological Dysfunction**	Clinical evaluation for evidence of depression, anxiety, OCD, PTSD, etc.
**9. Neurological Dysfunction**	Clinical examination, EMG, Small fiber biopsy, MRI brain, etc.
**10. Endocrine Abnormalities**	Laboratory evaluation of hormone levels (thyroid, adrenal, sex hormones, Vitamin D) and hormone precursors (DHEA-S, pregnenolone)
**11. Sleep Disorders**	Clinical evaluation (diet, medication), sleep studies, laboratory evaluation of hormone levels, etc.
**12. Autonomic Nervous System Dysfunction**	Tilt table testing with or without small fiber biopsies and autonomic/electrodiagnostic testing (EMG), clinical evaluation sitting/standing BP/heart rate
**13. Gastrointestinal Dysfunction**	Endoscopy, colonoscopy, clinical/laboratory evaluation (celiac markers, H. pylori), Comprehensive Digestive Stool Analysis (CDSA) for bacteria (*C. difficile*), ova and parasites, Candida, etc.
**14. Elevated Liver Enzymes**	Laboratory evaluation of AST, ALT, Alkaline phosphatase, total bilirubin, etc.
**15. Pain Syndromes**	Clinical evaluation, EMG, small fiber biopsy, laboratory markers for autoimmune disease (anti-myelin antibodies), etc.
**16. Deconditioning**	Clinical evaluation and need for physical therapy

**Table 2:** Frequencies and percentages of low, normal, and high immunoglobulin levels.

	N *	Low		Normal		High	
		*Frequency*	*Percent*	*Frequency*	*Percent*	*Frequency*	*Percent*
**IgA**	170	27	15.88	139	81.76	4	2.35
**IgM**	171	33	19.30	115	67.25	23	13.45
**IgG**	175	36	20.57	131	74.86	8	4.57
**SubClass1**	163	45	27.61	115	70.55	3	1.84
**SubClass2**	164	30	18.29	126	76.83	8	4.88
**SubClass3**	164	51	31.10	112	68.29	1	0.61
**SubClass4**	164	14	8.54	142	86.59	8	4.88

Frequencies and percentages of low, normal, and high immunoglobulin levels. A sizeable number of patients demonstrated immunoglobulin deficiencies and/or subclass deficiencies. Ranges for low, normal, and high values were determined by the reference laboratory. * Not every patient was tested for immunoglobulin and/or subclass deficiencies.
